# Contribution of Lateral Gene Transfers to the Genome Composition and Parasitic Ability of Root-Knot Nematodes

**DOI:** 10.1371/journal.pone.0050875

**Published:** 2012-11-30

**Authors:** Julien Paganini, Amandine Campan-Fournier, Martine Da Rocha, Philippe Gouret, Pierre Pontarotti, Eric Wajnberg, Pierre Abad, Etienne G. J. Danchin

**Affiliations:** 1 Aix-Marseille Université, Centre National de la Recherche Scientifique, LATP, UMR 7353, Evolution Biologique et Modélisation, Marseille, France; 2 INRA, Institut National de la Recherche Agronomique, UMR 1355 ISA, Institut Sophia Agrobiotech, Sophia-Antipolis, France; 3 CNRS, Centre National de la Recherche Scientifique, UMR 7254 ISA, Institut Sophia Agrobiotech, Sophia-Antipolis, France; 4 Université de Nice Sophia Antipolis, Institut Sophia Agrobiotech, Sophia-Antipolis, France; Universidade de São Paulo, Brazil

## Abstract

Lateral gene transfers (LGT), species to species transmission of genes by means other than direct inheritance from a common ancestor, have played significant role in shaping prokaryotic genomes and are involved in gain or transfer of important biological processes. Whether LGT significantly contributed to the composition of an animal genome is currently unclear. In nematodes, multiple LGT are suspected to have favored emergence of plant-parasitism. With the availability of whole genome sequences it is now possible to assess whether LGT have significantly contributed to the composition of an animal genome and to establish a comprehensive list of these events. We generated clusters of homologous genes and automated phylogenetic inference, to detect LGT in the genomes of root-knot nematodes and found that up to 3.34% of the genes originate from LGT of non-metazoan origin. After their acquisition, the majority of genes underwent series of duplications. Compared to the rest of the genes in these species, several predicted functional categories showed a skewed distribution in the set of genes acquired via LGT. Interestingly, functions related to metabolism, degradation or modification of carbohydrates or proteins were substantially more frequent. This suggests that genes involved in these processes, related to a parasitic lifestyle, have been more frequently fixed in these parasites after their acquisition. Genes from soil bacteria, including plant-pathogens were the most frequent closest relatives, suggesting donors were preferentially bacteria from the rhizosphere. Several of these bacterial genes are plasmid-borne, pointing to a possible role of these mobile genetic elements in the transfer mechanism. Our analysis provides the first comprehensive description of the ensemble of genes of non-metazoan origin in an animal genome. Besides being involved in important processes regarding plant-parasitism, genes acquired via LGT now constitute a substantial proportion of protein-coding genes in these nematode genomes.

## Introduction

Recognized cases of lateral gene transfers (LGT) in animals are relatively scarce compared to the plethora of examples in prokaryotes. Furthermore, most reported cases of gene transfers from a non-metazoan donor species to an animal host genome are not clearly linked to an identified biological process or life trait in the receiver species. Thus, it is difficult to assess whether LGT events have played an important evolutionary role in animal genomes [1]. Nevertheless, a few studies have shown an evident role of transferred gene products in the receiver animal organisms [2], [3], [4]. These examples involve gene transfers from non-metazoan eukaryotes to animals but not from prokaryotes to animals. This is surprising because much more cases of transfers from bacteria to animals than between eukaryotes have been reported so far [5]. Yet, several cases of LGT of bacterial origin and with significant functional consequences in animal have been reported from plant-parasitic nematodes. These nematodes represent an important economic threat as they are annually responsible for over 100 billion Euros loss in crop plants yields [6]. A recent review showed that genes acquired via LGT in these nematodes are involved in key parasitism processes such as modulation of plant defense, establishment of a feeding structure or degradation of the plant cell wall [7]. For instance, a whole repertoire of genes for the degradation of the plant cell wall has been acquired by several independent LGT events from different bacterial sources, followed by gene duplications [8]. Cases of LGT in plant-parasitic nematodes have been so far essentially identified indirectly by searching candidate parasitism genes and do not result from systematic and comprehensive genome scans. Consequently, there is currently no estimation of the total contribution of LGT to the genome composition and biology of plant-parasitic nematodes. Root-knot nematodes (*Meloidogyne* genus) are the most widespread and damaging of these plant parasites. A previous analysis of ESTs from three root-knot nematodes, *Meloidogyne javanica*, *M. incognita* and *M. hapla*, and comparison to the genomes of *C. elegans* and *D. melanogaster*, has provided a first “without *a priori*” overview of putative LGT events in these nematodes [9]. However, EST data only offer a fractional representation of the whole set of protein-coding genes in a given species and many potential LGT events may be missed in the absence of an available whole genome sequence.

Here, taking advantage of the availability of two root-knot nematodes whole genome sequences [10], [11], we have systematically searched potential LGT events of non-metazoan origin using a comparative genomics analysis with 16 species coupled with an automated phylogenetic reconstruction and tree topology scan. Using a phylogenetic approach not only allowed confirming similarity-based prediction of LGT but also examining the fate of genes after their transfer, including their duplication pattern. Our approach allowed retrieving all cases of LGT of non-metazoan origin in root-knot nematodes reported so far in the literature as well as new candidate cases of LGT not identified before. An analysis of the domain composition and putative functions of these genes indicates that they are preferentially involved in functions related to degradation, modification and metabolism of carbohydrates and proteins, reflecting the parasitic lifestyle of root-knot nematodes. Examination of the topologies of phylogenetic trees showed that the majority of genes acquired via LGT underwent series of duplications after their transfer. Overall, we show that up to 3.34% of protein-coding genes originate from LGT of non-metazoan origin in a root-knot nematode genome. Genes acquired via LGT do not appear to form clusters in the genome but the density of transposable elements is higher around genes acquired via LGT. Bacterial genes, including from notorious plant-pathogens sharing the same hosts as root-knot nematodes were frequently found as the most closely relatives. Finally, we discuss the hypothetical mechanisms involved in these LGT events and their evolutionary importance, both in the making of an animal genome and the emergence of plant-parasitism in nematodes.

## Results

### Identification of Lateral Gene Transfers in Root-knot Nematodes

**Figure 1:**
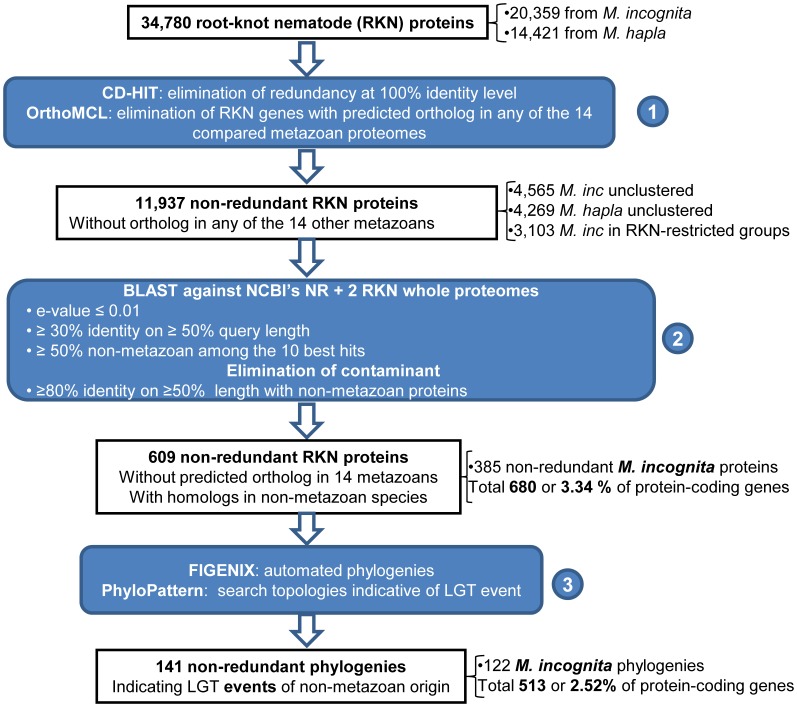
Schematic pipeline for detection of lateral gene transfers. This simplified representation highlights the three main steps used to detect potential lateral gene transfers of non-metazoan origin in root-knot nematodes. The three bioinformatics steps are represented within blue rectangles while initial, intermediate and final results are represented within white rectangles. Starting from 34,780 root-knot nematode proteins, step 1 consisted in eliminating redundancy at 100% identity and detecting orthologs in proteomes of 14 other metazoan species. Step 2 consisted in “blasting” all proteins that passed step 1 against the NCBI’s NR database completed with the whole proteomes of the two root-knot nematodes. Only proteins that returned at least 50% of non-metazoan hits among their 10 best blast hits were kept at this stage. Proteins that showed more than 80% identity with non-metazoan hits on at least half of their length were considered as contaminants and eliminated. All proteins passing step 2 were sent for automated phylogenetic analysis using FIGENIX pipeline. Topologies compatible with a lateral gene transfer were automatically searched among all generated trees using PhyloPattern. At the end of step 2 and of step 3, the total number of *M. incognita* protein-coding genes of non-metazoan origin, (including gene duplicates) and the proportion of the whole gene set are indicated in bold.

From pooled root-knot nematode whole proteomes (14,421 proteins from *M. hapla* and 20,359 from *M. incognita*), we identified a total of 11,937 non-redundant proteins that had no predicted ortholog in any of the 14 other compared metazoan genomes, based on an OrthoMCL [12] analysis (Figure 1, Table S1). Although these genes might actually have no evident homolog in metazoan species, our selection of 14 metazoan proteomes from various lineages (Figure S1) cannot be considered as fully representative of the spectrum of diversity present in animals. Furthermore, the presence and degree of conservation of these proteins in non-metazoan species needs to be assessed. We thus compared the 11,937 proteins apparently specific from root-knot nematodes against the NCBI’s non-redundant (NR) database using BLASTp [13]. Proteins that returned no significant hit in NR with the parameters we have set (methods) were discarded from the analysis because it is not possible to state whether (i) they actually originate from LGT event of as yet unidentified source or (ii) represent over-predicted gene models or (iii) represent true orphan genes restricted to root-knot nematodes. Because we are interested in transfers of genes from non-metazoan species to root-knot nematodes, we specifically selected root-knot nematode proteins that returned at least 50% of non-metazoan hits among their 10 best blast hits (Figure 1). A total of 609 non-redundant proteins satisfied this criterion and were considered as potentially originating from LGT of non-metazoan origin (Table S2).

**Figure 2:**
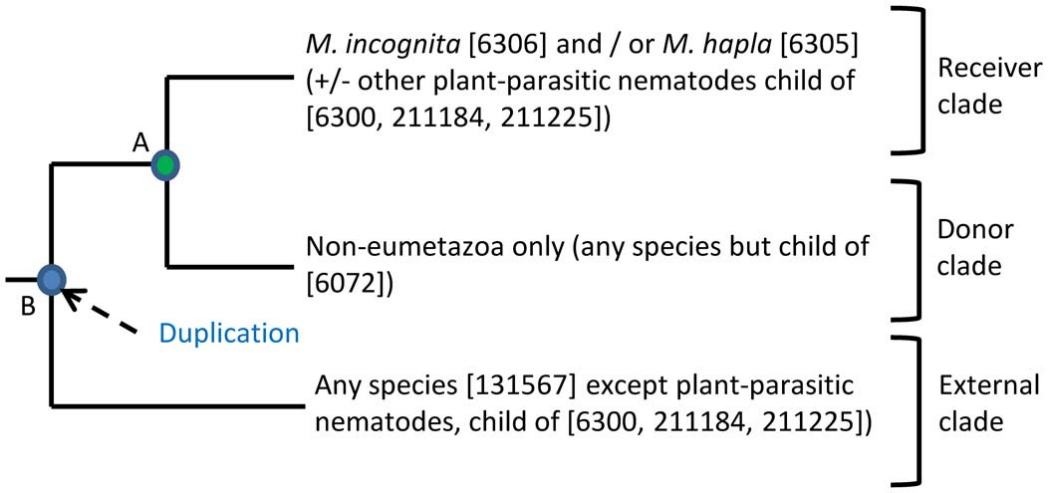
Phylogenetic pattern searched to identify lateral gene transfers. Schematic representation of the phylogenetic patterns searched with PhyloPattern [16] to identify trees harboring a topology indicating a lateral gene transfer of non-metazoan origin in root-knot nematode genomes. Basically, the topology searched is composed of three main clades. In every clade, species or taxonomic division authorized or forbidden as well as their NCBI’s taxonomy identifiers are indicated. The “receiver clade” must contain at least one sequence from *M. incognita* or from *M. hapla* and possibly from other species provided that these species are plant-parasitic nematodes. The “donor clade” can contain any species but eumetazoan (e.g. bacteria, fungi, plant, …). The external clade can contain any species but plant-parasitic nematodes. Presence of a node “A” connecting the receiver clade and the donor clade to the exclusion of the external clade is required and constitutes a minimal phylogenetic support for LGT. Strong support for LGT was assigned when, additionally, a node “B”, defined as follows was found. This node “B” must connect node “A” to the external clade and this node must be detected as a duplication node due to presence of at least one non metazoan species in the external clade.

These proteins were sent for automatic phylogenetic analysis using FIGENIX [14], [15] and topologies supporting potential lateral gene transfer events were searched using PhyloPattern within the DAGOBAH framework [16] (Figure 1, Figure 2). Phylogenetic trees were successfully constructed for 490 out of the 609 protein-coding genes. A total of 141 proteins yielded phylogenetic trees with topologies supporting LGT events (methods, Figure 2). The 141 trees can be consulted interactively in the I.O.D.A. database [17] (http://ioda.univ-provence.fr). The rest of the proteins (468) either did not return a tree topology compatible with the searched phylogenetic pattern(s) (349 cases) or due to an insufficient number of BLAST hit did not allow construction of a phylogenetic tree (119 cases). The corresponding genes were considered as possibly acquired via lateral gene transfer but without phylogenetic support (Table S2).

An analysis of the literature allowed us to establish a list of 15 distinct cases of genes or gene families acquisition via LGT in root-knot nematodes (Table 1). Interestingly, all these previously reported cases of LGT were retrieved in our systematic genome scan and all received a phylogenetic support. Hence, as a validation, our approach allowed retrieval of all previously published cases of candidate LGT events in *M. hapla* and *M. incognita*, indicating a good sensitivity. Concerning the specificity, we found all the previously reported cases within a set of only 141 non-redundant proteins that passed the BLAST filter and returned phylogenetic trees with topologies supporting LGT. Given that the number of root-knot nematodes proteins initially used in entry of the pipeline is 20,359 and 14,421 for *M. incognita* and *M. hapla*, respectively, our method can be considered highly specific.

**Table 1:** 15 distinct previously reported case of LGT in root-knot nematodes.

Gene/gene family	From ref.	Best phylogenetic support	RKN protein accession number
GH28 polygalacturonase	[7], [18], [39]	A+B	Minc18543b
PL3 pectate lyase	[7], [40], [41]	A+B	Minc01522c
GH43 candidate arabinase	[7], [10], [11]	A+B	Minc10639
GH5 cellulase	[7], [10], [18], [42], [43], [44]	A+B	Minc18711
GH30 xylanase	[7], [10], [18], [45]	A+B	Minc18650
Expansin-like protein	[7], [41]	A	Minc10987
GH32 candidate invertase	[7], [10], [18]	A	Mh_Contig1358:39..3354
Chorismate mutase	[7], [10], [11], [46]	A	Minc10536
Cyanate lyase	[7], [11]	A+B	Minc06015
VB5 pantothenate	[7]	A+B	Minc14603
VB7 biotin	[7]	A+B	Minc09512+ Minc09513*
NodL	[7], [9]	A+B	Mh_Contig222:75571..76264
MI00426 Glutamine synthetase (GSI)	[9]	A	Minc08077
MI01644 L-threonine aldolase	[9]	A+B	Mh_Contig2499:3012..6351
MI00109 candidate phosphoribosyltransferase	[9]	A	Minc16723

* Gene models Minc09512 and Minc09513 have to be fused to form a full length protein.

### Functions of Genes Acquired via LGT

In each of the two whole root-knot nematode proteomes, half of the proteins have been assigned at least one Pfam domain (50.9% and 49.59% for *M. incognita* and *M. hapla*, respectively). Based on these domains assignments, a total of 6,881 and 4,673 proteins were associated at least one Gene Ontology (GO) term, in *M. incognita* and *M. hapla*, respectively (Methods). All the GO terms were mapped to at least one parent term in the generic GO-slim ontology (Table S3). The distribution of GO terms for the three ontologies (biological process, cellular component and molecular function) were very similar between *M. incognita* and *M. hapla* whole proteomes, indicating a similar global qualitative distributions of putative functions in these two species despite different gene numbers (Table S3). The higher number of protein-coding genes in *M. incognita* compared to *M. hapla* is due to the peculiar structure of the *M. incognita* genome, mainly composed of pairs and triplets of similar yet divergent regions [10], [18]. The similarity of GO-terms distribution in *M. incognita* and *M. hapla* suggests that the frequency of gene copy retention in *M. incognita* has been homogeneous across the different functional categories.

**Figure 3:**
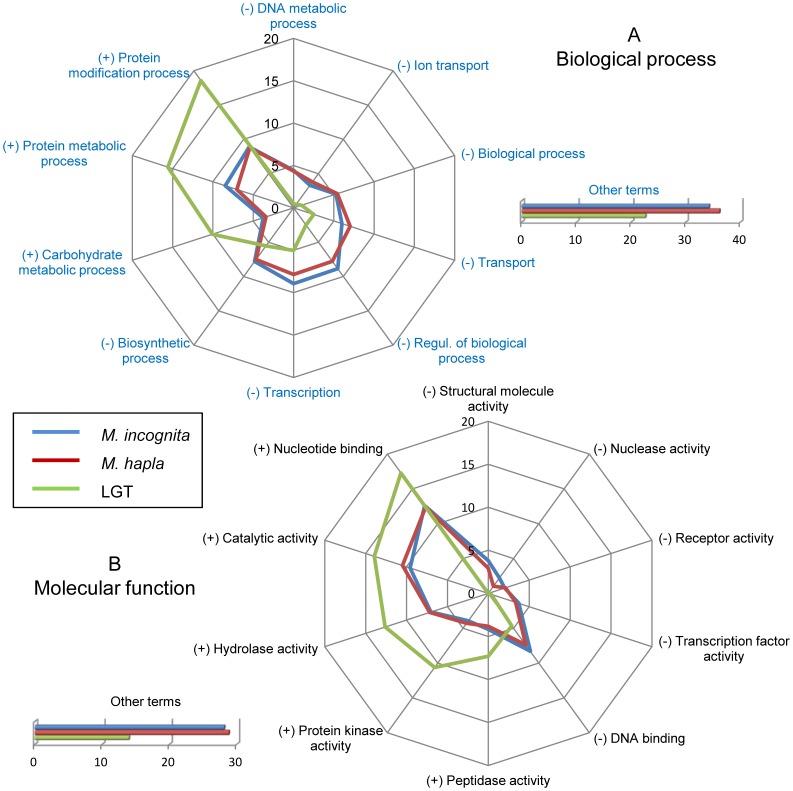
Functional categories with deviating abundance in the set of proteins acquired via LGT events. Kiviat diagram representing, the relative abundance of Gene Ontology (G.O.) terms, in percent for the whole *M. incognita* and *M. hapla* proteomes as well as for the 609 non-redundant root-knot nematode proteins originating from lateral gene transfer events. Distribution of G.O. terms in *M. incognita* and *M. hapla* are represented in blue and red, respectively. Distribution of G.O. terms in proteins acquired via LGT is represented in green. (A) Relative abundance of the G.O. terms assigned to root-knot nematode and LGT proteins in the Biological Process category. (B) Relative abundance of the G.O. terms assigned to root-knot nematode and LGT proteins in the Molecular Function category. In the two categories, the ten G.O. terms that presented the most different relative abundance (in percent) in LGT-acquired proteins in comparison to the whole root-knot nematode proteomes are presented.

We more specifically compared the distributions of GO-terms assigned to the 609 non-redundant candidate LGT proteins that passed both the OrthoMCL and BLAST filters to those of the whole root-knot nematode proteomes. Out of these 609 candidate LGT proteins, 335 (∼55%) were assigned at least one Pfam domain. Corresponding gene ontology terms could be assigned to 234 of these proteins (∼38% of the candidate LGT). For the three ontologies, the distributions of GO terms of the candidate LGT proteins were substantially different from those of the whole root-knot nematode proteomes (Figure 3, Table S3).

In the ‘biological process’ ontology, differences of highest amplitudes included an over-representation of the ‘carbohydrate metabolic process’, ‘protein metabolic process’ and ‘protein modification process’ terms in the set of LGT-acquired genes compared to the whole proteomes (Figure 3A ∼10% vs. ∼3–4% of annotated proteins for ‘carbohydrate metabolic process’, ∼16% vs. 7–8% for ‘protein metabolic process’ and ∼18% vs. ∼9% for ‘protein modification process’). In the ‘carbohydrate metabolic process’ category, several genes previously reported as acquired via LGT and known to encode plant cell wall-degrading or modifying enzymes were retrieved, including 12 GH5 cellulases, 3 GH28 polygalacturonases and 2 GH43 candidate arabinanases (Table S2). Besides these known LGT cases, other enzymes not previously described and potentially targeting plant polysaccharides were identified, including a putative starch-binding CBM20-bearing protein, a mannose 6p isomerase that can be involved in modification of the plant cell wall or a GH25 enzyme annotated in Pfam as possibly active on cell wall macromolecules. The ‘protein metabolic process’ category contained a majority of peptidases of different families, the most abundant being lon proteases that belong to MEROPS peptidase family S16. Phylogenetic trees indicate putative fungal and bacterial origins for these peptidases. Although these enzymes may be involved in degradation of plant proteins including for detoxification none has yet been experimentally characterized so far. Finally, the ‘protein modification process’ category mainly consisted of protein kinases. None had previously been reported and all those supported by a phylogeny indicate a candidate protist origin. Although 6 protein kinases have a predicted signal peptide and could be secreted by the nematodes, their precise role remains to be determined. In contrast, the term ‘regulation of biological processes’ was under-represented in the set of candidate LGT genes (∼3% vs. ∼8% of annotated proteins). Thus, overall, it appears that proteins with putative functions involved in carbohydrate metabolism as well as in protein metabolism and modification are over-represented in the set of proteins putatively acquired via LGT.

In the ‘molecular function’ ontology, we noted an over-representation of the protein kinase (∼11% vs. ∼4%), hydrolase (∼13% vs. ∼7%), catalytic (14% vs. 10%) and peptidase (7% vs. 4%) activities in candidate LGT-acquired proteins compared to the whole proteomes (Figure 3B). This reinforces and mirrors the over-representation of proteins involved in carbohydrate/protein degradation and metabolic processes in the “biological process” ontology. Curiously, we also found a slight over-representation of the ‘nucleotide binding’ term (17% vs 12%) but this was essentially due to the abundance of ATP-dependent protein kinases and peptidases in the LGT set. In contrast, we noted an under-representation of proteins annotated as ‘transcription factor’ (<0.5% VS ∼3%) or ‘regulation of biological processes’ (3% vs. 8%) indicating that these are not frequent functions of genes acquired via LGT or retained after transfer in these nematodes. In the ‘cellular component’ ontology, we remark a clear over-representation of proteins annotated as present in the ‘extracellular component’. Almost 30% of annotated proteins in the set of candidate LGT are annotated as such while only ∼2% of proteins are predicted to be in the extracellular component in the whole root-knot nematode proteomes (Table S3).

### Genes Acquired via LGT are Prone to Duplications

A previous phylogenetic analysis of genes encoding cell wall-degrading enzymes in plant-parasitic nematodes has shown that several genes underwent duplications after their acquisition via LGT and now form multigene families [8]. The same analysis showed that most of the duplications started before the separation of the different nematode lineages and, at least in root-knot nematodes, gene duplications have continued independently in the genomes of *M. incognita* and *M. hapla* after their separation from a common ancestor. In order to assess whether such a pattern of duplications is frequent after acquisition of a gene via LGT, we analyzed the 141 phylogenies indicative of an LGT. Out of these 141 phylogenies, 92 contain genes both from the *M. incognita* and *M. hapla* genomes, indicating they have been acquired at least in a common ancestor of the two nematodes (Table S4).

Using PhyloPattern (methods), we searched genes that underwent duplications since their acquisition in a common ancestor of the two root-knot nematodes. We found that after their acquisition, 76 of these genes (83%) underwent duplications either in one, both or a common ancestor of the two nematodes. Interestingly, in 79% of cases (60 out of 76), duplications have started before the separation of *M. incognita* and *M. hapla* and thus occurred in a common ancestor of the two species (Table S4). Duplications continued independently after the separation of the two lineages in 43 cases out of 60 (72%). In contrast, in no more than 16 cases, duplications occurred only after the separation of the two lineages in one or both Meloidogyne species. This observation indicates that the vast majority of genes acquired via LGT underwent duplications and most of these duplications (79%) started early in a common ancestor of the species.

We also assessed whether, at a large scale, and regardless phylogenetic support, genes putatively acquired via LGT have a higher tendency for species-specific duplications that the rest of protein-coding genes in the genomes of root-knot nematodes. We analyzed results of the OrthoMCL clustering to determine the number of species-specific duplications or in-paralogs (methods). Out of the 609 non-redundant genes putatively acquired via LGT, 403 were not clustered in any OrthoMCL group. These genes are thus present as single copies specific from *M. incognita* or from *M. hapla*. Out of the 206 remaining LGT genes in OrthoMCL groups, a total of 149 groups contain at least two genes from *M. incognita* or at least two from *M. hapla*. We discarded 26 LGT candidates present as two copies in *M. incognita* while in one single copy in *M. hapla*. Indeed, these copies might result from the genome structure of *M. incognita* and were not considered as having undergone “true” species-specific duplications. Overall, a total of 123 candidate LGT genes were present in at least three copies in *M. incognita* or at least two copies in *M. hapla* (Table S5). Hence, 59.71% of the 206 LGT genes present in OrthoMCL groups have undergone species-specific duplications since the separation of the *M. incognita* and *M. hapla* lineages from their common ancestor. Duplications ranged from multigene families of size 2 to 25 in a single species (Table S5). While some genes underwent duplications both in *M. incognita* and *M. hapla* after their separation from a common ancestor, most gene duplications observed were asymmetric. For instance, LGT gene Minc09058 is present in 25 copies in *M. incognita* and no ortholog was found in *M. hapla* (Table S5). In contrast, LGT gene Minc18743 is present in one single copy in *M. incognita* while it is present in 6 copies in *M. hapla*. Hence, there are no systematic tendencies for a given gene to be equally duplicated and fixed in both root-knot nematode species. To assess whether the proportion of lineage-specific duplication is different for LGT genes than for the remainder of the genes in root-knot nematodes, we calculated the number of in-paralogs in the whole genome of *M. incognita*. In this nematode, 20,359 gene models have been predicted [10] and 15,365 genes are present in 7,647 OrthoMCL groups. As for LGT genes, we discarded groups containing *M. incognita* single-copy genes and those containing two copies in *M. incognita* but a single copy in *M. hapla*. Overall, a total of 2,137 OrthoMCL groups out of 7,647 (27.94%) contain at least three *M. incognita* in-paralogs and represent species-specific duplications. In comparison, in the set of genes acquired via LGT, the proportion of OrthoMCL groups with in-paralogs is more than twice as high. This observation suggests that genes acquired via lateral transfer are prone to duplications that continue independently in different species after their acquisition in a common ancestor.

### Contribution of LGT of Non-metazoan Origin to the Genome of a Root-knot Nematode

In root-knot nematodes, we identified a total of 609 non-redundant genes with no predicted ortholog in 14 other metazoan species and that returned more than 50% non-metazoan hits in blast searches. Because a majority of genes underwent duplications after their acquisition, the estimation of their total abundance in extant genomes has to take duplications into account. Out of the 609 non-redundant genes acquired via LGT, a total of 385 are from *M. incognita* and 202 of these do not cluster in any OrthoMCL group. These genes are thus present in single copy in *M. incognita* and are absent in the 14 other metazoan species compared. In contrast, 183 *M. incognita* genes are in OrthoMCL groups with at least another gene either from *M. incognita* or from *M. hapla*. Considering that *M. incognita* genes present in multiple copies in a same OrthoMCL group underwent duplications after their acquisition via LGT, the total number of genes of non-metazoan origin in *M. incognita* is 680 (202 singletons and 478 gene copies present in 183 groups, Table 2). This represents ∼3.34% of the 20,359 protein-coding genes in *M. incognita*.

**Table 2:** Total number of LGT genes in the genome of *M. incognita.*

	OrthoMCL+Blast		Phylogenetic inference	
Copies	# MCL Groups	# Genes	# trees	# Genes
1	65*	267**	34	34
2	59	118	27	54
3	23	69	10	30
4	16	64	9	36
5	6	30	7	35
6	2	12	2	12
7	5	35	3	21
8	2	16	2	16
>8	5	69	28	275
**Total**	**183**	**680**	**122**	**513**

* 65 groups containing a single *M. incognita* protein and at least one *M. hapla* gene.

** 65 genes in OrthoMCL groups + 202 single copies that were not clustered in any OrthoMCL group.

We also estimated the proportion of genes of non-metazoan origin that, besides OrthoMCL and Blast support, also received phylogenetic support for LGT. Out of the 141 non-redundant phylogenies compatible with LGT in root-knot nematode, 122 contain at least one *M. incognita* gene in the LGT subtree. To account for gene duplications after acquisition via LGT, we counted the total number of *M. incognita* genes per acceptor subtree. Overall, we enumerate a total of 513 or 2.52% of *M. incognita* protein-coding genes with phylogenetic support for acquisition via LGT (Table 2, Table S4).

Hence, we estimate that genes of non-metazoan origin represent between ∼2.52% and ∼3.34% of protein-coding genes in a root-knot nematode, depending on whether or not phylogenetic support is required.

### Distribution of LGT Candidates Along a Root-knot Nematode Genome

Considering duplications after transfer, in the genome of *M. incognita*, as much as 680 genes are of non-metazoan origin. We analyzed the distribution of these genes on the 2,817 *M. incognita* scaffolds and showed that 38 clusters contain three or more putative LGT genes separated by no more than 50 kb, representing a total of 161 LGT candidates (Table S6). The five largest clusters contain 16, 8, 7, 7 and 6 genes putatively acquired via LGT on scaffolds 90, 53, 85, 91, and 69, respectively (Table S6). One of the largest clusters comprises 7 genes encoding pectate lyases of family PL3 [19] on scaffold 53. Interestingly, a similar cluster, consisting of 4 genes encoding PL3s was found in the genome of *M. hapla* [11], suggesting that they derive from a common ancestral cluster that predates the separation of *M. incognita* and *M. hapla* lineages. The five largest clusters all consist of multiple copies of a same or a few different genes. This is attested by membership to the same OrthoMCL groups and/or a same Pfam domain annotation. The largest cluster in *M. incognita*, consists of 16 genes present on scaffold 90. This cluster is no exception to the rule and these 16 genes belong to only 6 distinct OrthoMCL groups and 14 have the same predicted Pfam Kinase domain.

The longest cluster containing only different genes putatively acquired by LGT consists of 5 genes on scaffold 154. The five genes have different predicted Pfam domains and none belong to a same OrthoMCL group. However, only two out of the five genes have a phylogenetic support for possible LGT. Hence, overall, the vast majority of clusters of candidate LGT genes consist of copies of a same or a few distinct genes not of aggregation of multiple independently-acquired genes from distinct families.

### Putative Donors for LGT are Mainly Soil Bacteria

From the 141 phylogenetic trees indicative of an LGT event, we reported the ensemble of species present in the putative donor sub-trees. Donor sub-trees consist in monophyletic groups composed exclusively of non-metazoan species and holding the closest outgroup position relative to the plant-parasitic nematode receiver group.

**Figure 4:**
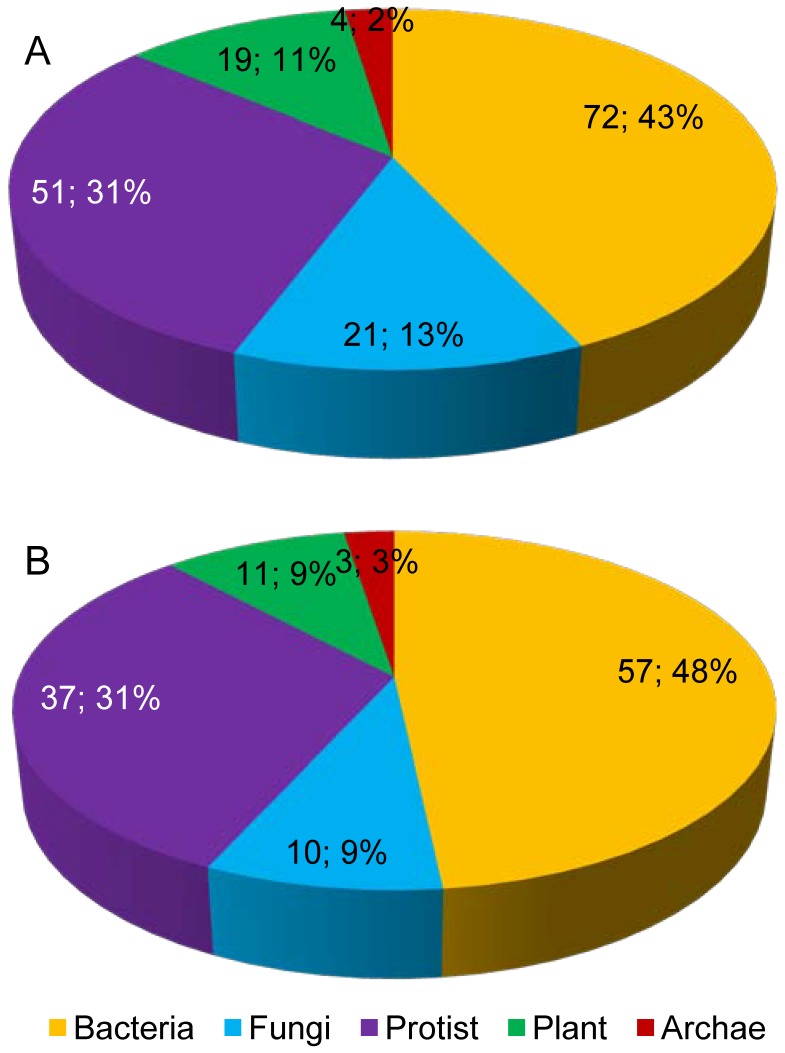
Taxonomic distribution of candidate donors for LGT events. (A) Number and proportion of phylogenetic trees that contain at least one species of a given taxonomic division in the closest donor clade. (B) Number and proportion of phylogenetic trees that contain only species of a given taxonomic division in the closest donor clade. The different possible taxonomic divisions are Bacteria, Fungi, Protists, Plants and Archaea. Note that Protist is not a monophyletic division and encompasses several distinct eukaryotic lineages.

Bacteria were present in donor sub-trees in 72 out of the 141 phylogenetic trees and represented the most frequent taxonomic division. In 57 cases the donor sub-tree contained only bacteria while in 15 cases, species from other kingdoms were present besides bacteria (Figure 4, Table S7). Interestingly, in many occasions, bacterial species found in the donor clades are soil bacteria, including notorious plant pathogens (e.g. *Ralstonia solanacearum*, *Xanthomonas oryzae*, *Xanthomonas campestris, Pseudomonas syringae*) plant symbionts (e.g. *Sinorhizobium meliloti*, *Methylobacterium nodulans, Mesorhizobium loti*), or more generally species known to dwell in the rhizosphere, the region of soil surrounding plant roots (e.g. *Burkholderia ambifaria*, *Agrobacterium radiobacter*, *Flavobacterium johnsoniae*).

The next most frequent category in the donor sub-trees is protist, a non-monophyletic group of mostly unicellular eukaryotic microorganisms. In 51 out of the 141 phylogenetic trees, protists were present in the donor sub-tree. In 37 cases protists were present alone whereas in 14 cases they were associated with species from other kingdoms. Ciliophora, amoebozoa, apicomplexa and oomycetes were the most frequently present protists. Interestingly, while Oomycetes were found in donor sub-trees in five distinct cases, this is systematically due to one single species, *Phytophtora infestans*, a known plant-pathogen. Similarly, some of the Amoebozoa found in donor sub-trees are known soil-dwelling slime molds (e.g. *Polysphondilium palladium*, *Dictyostelium discoideum)*. For Ciliophora, in contrast, most retrieved species dwell in freshwater although some of them have a more ubiquitous habitat, including soil. Finally, in the apicomplexa category, mainly animal parasites are found (e.g. *Babesia bovis*, *Toxoplasma gondii*). However, any conclusion on the habitat of these species must be taken with caution because the biodiversity in protists has been so far much less extensively sampled than those of bacteria and fungi.

Fungi were identified in the donor clades in a total of 21 out of 141 trees and represent in fact the second most frequent monophyletic category. In 10 cases, only fungi are found in the donor clade while in 11 cases, fungi are found associated with species from different kingdoms. Among the fungal species identified in the donor clades, many notorious plant pathogens were found (e.g. *Magnaporthe grisea*, *Sclerotinia sclerotiorum, Botrytis cinerea*) as well as soil-dwelling fungi (e.g. *Chaetomium globosum, Aspergillus fumigatus*).

Plants were also identified as potential donors in 19 phylogenetic trees. In 11 cases the donor sub-tree contained only plants while association with species from other kingdoms was observed in 8 cases. Plant species identified ranged from the unicellular green alga *Chlamydomonas reinhardtii* that possesses a ubiquitous habitat, including soil, to plant that are compatible hosts of root-knot nematodes (e.g. *Oryza sativa*, *Nicotiana attenuata*).

Finally, we also identified Archaea in donor clades of 4 phylogenetic trees and 3 of these clades contained only Archae. All archaeal species identified were hyperthermophilic and thus do not share an evident common habitat with root-knot nematodes. Though, once again the sampled biodiversity in Archea is not deep enough to allow concluding on the habitat of the potential donors.

### Several LGT Genes Crucial for Parasitism have Homologs on Bacterial Plasmids

Mobile genetic elements such as plasmids or bacteriophages are commonly involved in LGT events between bacteria. Because bacteria have been frequently found in the donor sub-trees in our analysis, it is interesting to search whether some of the genes putatively acquired via LGT in nematodes are present on microbial mobile genetic elements. Out of the 609 non-redundant candidate LGT genes in root-knot nematodes, a total of 146 returned blast e-values <0.001 with proteins present on microbial mobile genetic elements (methods). Overall, 117 of these protein-coding genes (80%) were found on known bacterial plasmids while 19 were found on prophages and 10 on bacterial viruses (Table S8A). We further focused our analysis on 32 root-knot nematode proteins that aligned with at least 30% identity on at least half of their length with proteins present on microbial mobile elements (Table S8B).

Out of these 32 proteins, 28 are plasmid-borne in candidate donor bacteria. Interestingly, among the plasmid-borne proteins, 9 are present in the list of 15 previously reported clear cases of LGT from the literature (Table S8B, Table 1). These 9 proteins encompass a Chorismate mutase, thought to be involved in modulation of plant defense, aVB5 pantothenate, aVB7 biotin, and a GH32 candidate invertase all probably involved in nutrient processing, a NodL suspected to play a role in the establishment of the root-knot nematodes feeding structure, two GH30 xylanases involved in degradation of plant polysaccharides [7] and a candidate phosphoribosyltransferase as well as a candidate L-threonine aldolase both of as yet unknown function in nematodes [9]. Hence, several genes that, according to the literature, play important functions in plant-parasitism have homologs borne by bacterial mobile elements.

For the 32 genes putatively transferred via plasmid or prophage vectors, a total of 21 different potential donor bacteria are found. Interestingly and in line with the previous section on potential donors, 16 of these bacterial species are known to dwell in the rhizosphere (e.g. *Ralstonia or Rhizobium*), the same habitat than root-knot nematodes (Table S8B).

### The Density of Transposable Elements is Higher in the Vicinity of Genes Acquired via LGT

**Figure 5:**
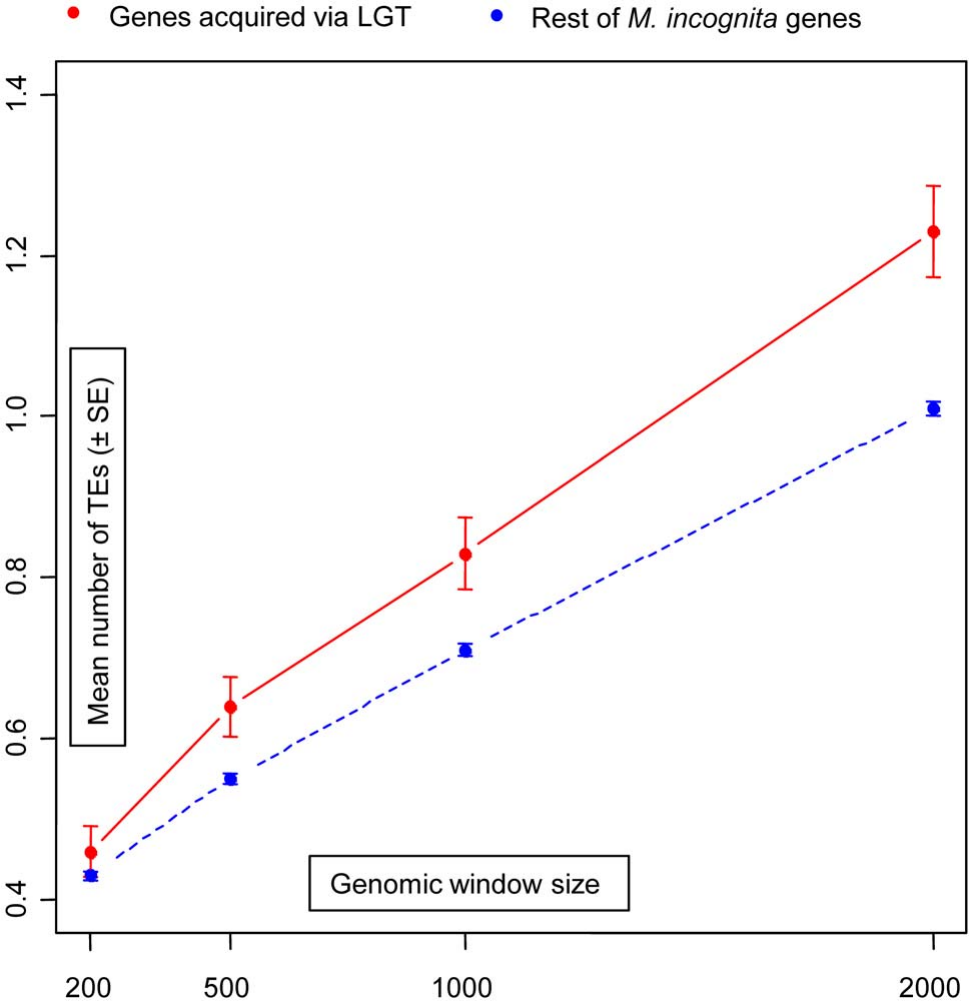
Density of transposable elements around genes acquired via LGT in *M. incognita*. Plot of the mean number of transposable elements (± standard error) in genomic windows of size 200, 500, 1,000 and 2,000 bp around genes acquired via LGT (red) and around the rest of protein-coding genes (blue) in the genome of *M. incognita*.

Transposable elements (TEs) can jump from one position to another in a genome as well as between the genomes of different species across the kingdom boundaries and are even known to mediate transfer of genes within a species genome through a hitchhiking-like process [20]. Previous report of LGT to metazoan receiver species, in the bdelloid rotifer *Adineta vaga* [21] and in the necromenic nematode *Pristionchus pacificus* [22] have both shown a genomic environment rich in transposable elements (TEs) around genes acquired via LGT. To evaluate the density of TEs around LGT genes in root-knot nematodes, we counted the number of TEs present in genomic windows of size 200, 500, 1,000 and 2,000 bp around genes acquired via LGT and around the rest of protein-coding genes in the *M. incognita* genome (methods). We found that the density of TEs was significantly higher around genes acquired via LGT for all genomic windows of size ≥500 (Table 3, Figure 5). Hence, similarly to *A. vaga* and *P. pacificus*, the genomic environment around LGT genes is rich in TEs in root-knot nematodes.

**Table 3:** Density of transposable elements around genes acquired via LGT and around the rest of protein-coding genes in *M. incognita.*

Window size	Gene type	Mean # of TE in window	Std error	X^2^ (1 df)*	p-value
200	LGT	0.46	0.032	1.05	0.306
	Other	0.43	0.006		
500	LGT	0.64	0.037	5.96	0.015
	Other	0.55	0.007		
1,000	LGT	0.83	0.044	8.07	0.005
	Other	0.71	0.008		
2,000	LGT	1.23	0.057	17.27	<0.001
	Other	1.01	0.009		

* result of the chi-square test with one degree of freedom.

In the bdelloid rotifer *Adineta vaga*, genes acquired via LGT were more frequently found in telomeric regions rich in TEs [21]. We checked whether in the genome of *M. incognita*, transferred genes had a tendency to accumulate at the tips of scaffolds. Although with 2,817 scaffolds we are far from the estimated 30–40 chromosomes in *M. incognita*, we observed no sign for grouping of candidate LGT genes at scaffold ends.

### GC Content and Codon Usage

Because genes acquired via LGT originate from species that can feature codon usage and GC content markedly different from those of the receiver species, these genes might have kept characteristics of their genome of origin. For instance, in the necromenic nematode *P. pacificus*, genes acquired via LGT from insects have kept codon usage more closely related to those of insect donors than that of the “endogenous” *P. pacificus* genes [22].

**Figure 6:**
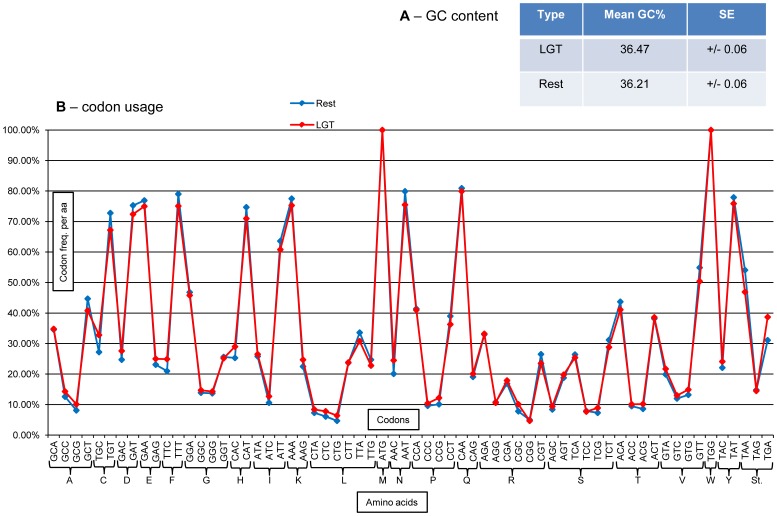
GC percent and codon usage of LGT genes in *M. incognita*. (A) Average and standard error (SE) percent of GC nucleotides in genes acquired via lateral gene transfer (LGT) and in the rest of protein-coding genes in *M. incognita* (Rest). (B) Comparison of the relative percent of codon usage per coded amino acids between genes acquired via lateral gene transfer (LGT) and the rest of protein-coding genes (Rest). X-axis: the 64 different codon and their associated 20 amino-acids and Stop. Y-axis: the percentage of usage of a given codon for a given encoded amino-acid.

We thus compared the codon usage and GC content of genes acquired via LGT in *M. incognita* to the rest of protein-coding genes (methods). With an average GC content of 31.4%, the *M. incognita* whole genome is globally GC poor. We measured an average GC content for a protein-coding gene in *M. incognita* of 36.21% (excluding genes acquired via LGT). By comparison, the average GC content for the 680 genes acquired via LGT is 36.47% (Figure 6A). We also generated codon usage tables for genes acquired via LGT and for the rest of the *M. incognita* gene set (Figure 6B, Table S9). The two codon usage tables were very similar with an average difference in frequency of codon usage for a given encoded amino-acid of 0.02 (2%). Only two sets of codons differed by more than 5% in frequency, Cystein codons and STOP codons.

Hence, genes acquired via LGT in *M. incognita* cannot be differentiated from the rest of protein-coding genes based on their GC content or codon usage.

## Discussion

Our analysis represents the first comprehensive pan-genomic search for LGT events in nematodes with phylogenetic validation. Previous reports have shown that different genes acquired by LGT in plant-parasitic nematodes play important roles in the parasitism process and hence, have been functionally significant [7]. Our systematic search for LGT events of non-metazoan origin overall confirms their link to functions important for parasitism but also provides the first estimate of the total contribution of LGT to the making of a metazoan animal genome.

### Contribution of LGT to the Genomes of Root-knot Nematodes

In a single root-knot nematode species, *M. incognita*, we have estimated that between 513 and 680 genes are of non-metazoan origin, depending on whether or not phylogenetic support is required. This represents between ∼2.52% and ∼3.34% of the protein-coding genes in this nematode, a substantial proportion. For comparison, this is more than the whole proportion of genes encoding carbohydrate-active enzymes (420 genes, 2.06%) or peptidases (334 genes, 1.64%) in the *M. incognita* genome [10].

To date, a whole genome inventory of genes acquired via LGT in a nematode has been established in only one other species, the plant-pathogenic agent of pine wilt disease, *Bursaphelenchus xylophilus*. Although no extensive functional analysis nor information of their genomic distribution and environment have been performed, this analysis revealed that between 24 and 223 genes were possibly acquired via LGT in this nematode, depending on whether phylogenetic or BLAST support were taken into account [23]. Representing between ∼0.13% and ∼1.25% of the 18,074 predicted protein-coding genes, the proportion in *B. xylophilus* is lower than in *M. incognita* but remains significant. We can expect that exploration of forthcoming genomes of other nematodes, including plant and animal parasites will bring new evidences for the importance of LGT in the making of a metazoan genome.

Although already representing a substantial proportion of protein-coding genes, the set of predicted LGT-acquired genes we present in our analysis is probably a minimal estimate of the actual number of such acquired genes. Indeed, we used stringent criteria in order to eliminate as much as possible false positives but necessarily may have missed some true positives. For instance, a gene that would have been transferred once in plant-parasitic nematodes and once in another metazoan would be either eliminated by our OrthoMCL filters or yield a phylogenetic tree not compatible with the searched LGT PhyloPattern. Similarly, we focused our analysis on transfers from non-metazoan donors to nematode receivers. Thus, we do not consider here transfers of metazoan origin in these nematodes. As unlikely as such event might appear, it was recently proposed that several genes in the necromenic nematode *P. pacificus* have been transferred from insect donors via LGT [22]. The total number of genes that do not originate from a common ancestor via vertical inheritance is thus probably even higher than currently estimated in plant-parasitic nematodes.

In this analysis, we have considered that genes have been transferred from non-metazoan donor to metazoan root-knot nematodes. Although it can be hypothesized that transfers may have occurred in the other direction (metazoan to non-metazoan), this appears substantially less likely for the following reasons. First, except root-knot nematodes and their close plant-parasitic relatives, no other nematode possess these genes while in the donor clades the diversity of represented phyla is generally high. Hence, this would require that the genes have been “invented” in root-knot nematodes then transferred to multiple non-metazoan species, independently, an unlikely hypothesis. Second, LGT genes all possess one or more spliceosomal introns and in many cases, closest relatives are bacterial. It intuitively appears more difficult to transfer an eukaryotic multi-exon gene in a bacterial species than an intronless bacterial gene in an eukaryotic genome followed by intron gains. Third, at least in the 52 phylogenetic trees that present a “duplication” node B, the direction of the transfer is explicitly from non-metazoan to metazoa because in these cases root-knot nematode genes are nested inside clades of non-metazoan species. In contrast, transfers in the other direction would require non-metazoan genes to be nested within plant-parasitic nematode clades.

### Contribution of LGT to the Biology of Root-knot Nematodes

Root-knot nematodes possess a spear-like structure named “stylet”, connected to esophageal and dorsal gland cells. Proteins secreted through this stylet in plant tissue are called effectors and play essential roles in the parasitic interaction [24]. Most gene products of LGT cases reported so far in root-knot nematodes are effectors. Our whole genome scan for LGT events allowed retrieving all the 15 distinct cases of LGT reported so far in the literature (Table 1). The effectors originating from these LGT events are involved in processes such as modulation of plant defense, establishment of a feeding structure, nutrient processing or degradation of the plant cell wall [7]. Besides these 15 known cases, all the other genes we detected as acquired via LGT had not been described previously in the literature and the possible function they play in root-knot nematodes has been so far unexplored. Search for Pfam protein domains and deduction of Gene Ontology terms allowed retrieving information on the putative functions for approximately 40% of the 609 non-redundant genes of non-metazoan origin. Compared to the rest of protein-coding genes in root-knot nematodes, we observed an over-representation of proteins putatively involved in carbohydrate metabolism, in protein metabolism and modification as well as in hydrolase, catalytic or peptidase activities. Functions related to carbohydrate and protein metabolism, catalytic and hydrolase activities point to degradation of the plant cell wall and nutrient processing. A possible role in detoxification can be proposed for sequences annotated with the “protein modification” term. We also remarked that proteins predicted to be secreted or in the extracellular compartment were more frequent in the LGT set.

This ensemble of observations suggests that genes acquired via LGT mainly encode proteins secreted by the nematode and involved in degradation of carbohydrates and proteins, in nutrient processing, metabolism and detoxification processes. Overall, these predicted functions make sense considering the plant-parasitic lifestyle of the root-knot nematodes and echoes the functional roles assigned to the previously reported cases of LGT in these species.

In contrast, we remarked that functions related to regulation of biological processes or transcription were under-represented in the set of genes acquired via LGT. Although there is no *a prior*’ reason for a functional category to be more prone to LGT than another, there are reasons for a category to be more easily fixed at the population level and then at a species level. Indeed, genes that provide a selective advantage through transfer of a new capability may have benefited from positive selection and consequently accelerated fixation. For a gene to be positively selected, it must first be functional in the receiver species. Genes involved in core basic biochemical functions, such as degradation of a carbohydrate or a protein, intuitively appear more likely to be able to perform the same exact function in a distant receiver organism than genes involved in processes such as fine regulation of gene expression or of biological processes in multicellular organisms.

### Duplications after Acquisition via LGT

Our analysis revealed a high propensity for duplications after acquisition of genes via lateral transfer. The majority of duplications we observed started before the separation of the *M. incognita* and *M. hapla* lineages and continued independently in the two root-knot nematode genomes. We had previously observed a comparable tendency for at least three gene families encoding plant cell wall-degrading enzymes in these nematodes [8]. Our whole genome analysis suggests the phenomenon is more general after an LGT event. Interestingly, importance of duplications, including some that started before the separation of the different nematode lineages analyzed and that continued independently after their separation has also been reported in necromenic nematodes of the *Pristionchus* genus [25]. The presence of LGT genes as multigene families suggests that positive selective pressure may have favored individuals with multiple copies of laterally-acquired genes. Observation that duplications constitute an adaptive mechanism to overcome a change or a stress in the environment at the time scale of a few generations has lead to propose an evolutionary model of adaptive radiation for the origin of new gene functions [26]. Under this model, duplication of sub-optimal genes may allow emergence of new gene variants with more optimal or divergent function through neo-functionalization [26]. Duplications can also allow specialization and partition of function through sub-functionalization. Interestingly, it has recently been shown that spontaneous gene duplications occur at a much higher rate than point mutations in *C. elegans*, suggesting that early adaptive genomic changes could be supported more by advantageous duplications than by beneficial mutations, in particular when dosage is under selective pressure [27]. In the case of an LGT event of non-metazoan origin, it is likely that the transferred gene was initially poorly adapted to the nematode genome’s GC content, codon usage and regulatory elements. Consequently, individuals with multiple copies of the transferred genes probably presented a higher probability for the emergence of a fully functional gene or for expression of the gene product at a sufficient level. Similarly, presence of the acquired gene in multiple copies may have favored the emergence of new functions. For this ensemble of reasons, individuals harboring multiple copies of genes acquired via LGT may have been positively selected generation after generation leading to the eventual presence of multigene families in root-knot nematode genomes after transfer of a single gene.

Overall, the most common fate of gene copies after duplication is their loss, often via pseudogenization [28]. In teleost fishes, it has been estimated that only 15% of gene copies were maintained in a functional form after whole genome duplication [29]. It is therefore potentially interesting to search pseudogenes in multigene families acquired via LGT. A recent analysis has allowed identification of candidate pseudogenes encoding altered cellulases acquired via LGT in the peanut pod nematode *Ditylenchus africanus* but no significant traces of pseudo-cellulases were found in the genomes of *M. incognita* and *M. hapla* [30]. This apparent absence of pseudogenes suggests that most genes present in multiple copies in extant Meloidogyne genomes have initially arisen from old duplications and have been fixed early; the possible old pseudogenes probably accumulated too much mutations since their initial decay to be differentiated from the intergenic DNA in present genomes.

### The Nature of Putative Donors and Possible Transfer Vectors

Although identification of the exact donor in an LGT event appears extremely challenging, analysis of the phylogenetic trees provides information on the nature of potential donors at the kingdom and phylum levels. Overall, we remarked that bacteria constituted the most frequent group of potential donors and many bacteria from the rhizosphere, including plant-pathogens and plant-symbionts were present in these donor clades. Similarly, a number of plant-pathogenic fungi were also identified in the donor clades. Species present in the rhizosphere share the same environment than root-knot-nematode and thus appear as interesting candidate donors, particularly because several of the genes known to have been acquired via LGT in these nematodes are involved in parasitic interactions with the plant.

Even if these species share a common environment and similar lifestyles, in association with plants, the mechanism of transfer itself remains elusive. In the case of plant cell wall-degrading enzymes, acquisition through feeding on plant-associated bacteria was favored over hypothesis of acquisition from bacterial endosymbionts. Indeed, no homologs of the genes acquired via LGT had been identified in known nematode endosymbiont while many are found on bacteria that are plant-parasites or plant-symbionts [31].

Regardless the origin, different mechanisms of transfer, including bacterial secretion systems or possible intermediates such as viruses, transposable elements or plasmids have been evoked [32]. Our whole genome analysis showed that the genomic environment around LGT genes is rich in transposable elements and that several bacterial homologs of the transferred genes are plasmid-borne.

It is tempting to propose that plasmids may have served as vectors for the transmission of genes of bacterial origin in the genome of the root-knot nematodes. The genes putatively transferred via plasmids include some previously characterized as important for parasitism processes, such as GH30 xylanases involved in the degradation of the plant cell wall or chorismate mutases involved in plant defense modulation. Plasmids are already known to support LGT and acquisition of new capabilities such as antibiotic resistance between bacteria. Our findings suggest that plasmids may have also played a significant role in the transfer of bacterial genes of functional importance to the genomes of root-knot nematode. The mechanism of transfer from bacterial plasmids to the genome of nematodes may be similar to the transfer of genes via Agrobacterium Ti plasmid to nuclear genome of plant cells. As for any case of putative lateral gene transfer, it is important to ascertain that genes do not result from a contamination. This hypothesis can be ruled out in our case for several reasons. (i) the 32 genes that present significant similarities with plasmid-borne bacterial genes have between one and 13 spliceosomal introns. (ii) the sequence similarities between these nematode genes and their bacterial plasmid counterparts range at best between 30–40% identity, far from the level expected for a contamination. (iii) these genes are assembled in the root-knot nematode genomes in the vicinity of true nematode genes presenting significant similarities with genes in *C. elegans*.

Similarly to reported cases of LGT in two other animals, the bdelloid rotifer *Adineta vaga* and the necromenic nematode *Pristionchus pacificus*, we observed a preferential distribution of LGT genes in regions rich in transposable elements (TEs) in root-knot nematode genomes. Thus, a possible role of TEs as hitchhiking vector in the mechanism of transfer can be hypothesized. Supporting this possibility, it has been shown that a DNA transposon has undergone repeated lateral transfers in different tetrapod species, including human [33]. An alternative hypothesis is that TEs do not play any role in the mechanism of transfer but that some regions in root-knot nematode genomes are more tolerant to both the accumulation of TEs and integration of genes of foreign origin.

### LGT Events have been Multiple and Probably Ancient

Analyses of our phylogenetic tree topologies have indicated that multiple different species and kingdoms were positioned in donor clades, suggesting that there is not a single or low number of donors but a multitude of possible species. Consistent with these observations we did not identify clear genomic clusters of different genes acquired via LGT. All the biggest LGT genomic clusters consist of repeats of a same or a few genes that underwent duplications after their acquisition via LGT. Overall, these features suggest that the clusters of LGT genes observed in the *M. incognita* genome result from multiple cis-duplications and not from “en-bloc” co-transfers from a same donor or from multiple independent transfers in a hotspot of integration of foreign genes. Previous observations on genes acquired via LGT and involved in degradation of the plant cell wall had also shown no evidence for clustering of different gene families in a same genomic region in *M. incognita* [8].

Overall, we remarked that genes acquired via LGT showed GC content and codon usage very similar to those of the other protein coding genes in *M. incognita* despite putative origins in a multitude of evolutionary distant donors. Based on these characteristics, they cannot be distinguished from typical endogenous root-knot nematode genes. This suggests that, in general, transfer events have been sufficiently ancient to have allowed adaptation to the codon usage and GC content of a typical *M. incognita* protein-coding gene. An alternative hypothesis is that the only gene transfers that have been successful are those that involve donor genes featuring GC content and codon usage similar to those of the receiver species. However, given the multitude and diversity of putative donors, this hypothesis appears unlikely. Furthermore, in the necromenic nematode *P. pacificus*, genes acquired via LGT of insect origin showed a codon usage more similar to those of insect donors than to those of the other nematode genes [22]. This indicates that there is no prerequisite in terms of similarity in codon usage or GC content for an LGT to occur.

### Conclusions

Overall, our root-knot nematode pan-genomic analysis shows that, even if LGT events are not as prevalent as in prokaryotes, they also have significantly contributed both to the genome composition and biology in these metazoan animals. Representing up to 3.34% of protein-coding genes, predicted and known functions of genes acquired via LGT indicate a clear link with different processes crucial for plant-parasitism. Hence, LGT events have probably played an important role in the emergence of this capability in nematodes. Further comprehensive whole genome search for LGT events in other metazoan species will probably allow assessing whether evolutionary and biological importance of LGT is a specificity of nematodes or whether the phenomenon is more general in metazoan species.

## Methods

### Determination of Groups of Orthologous Metazoan Proteins

The whole sets of predicted proteins from the root-knot nematodes *Meloidogyne incognita* and *Meloidogyne hapla* were compared to those of 14 other metazoan species using the OrthoMCL [12] software with default parameters. The 14 other metazoan species compared are *Branchiostoma floridae*, *Brugia malayi, Bombyx mori, Caenorhabditis briggsae, Caenorhabditis elegans, Ciona intestinalis, Drosophila melanogaster, Homo sapiens, Mus musculus, Nematostella vectensis, Pristionchus pacificus, Strongylocentrotus purpuratus, Trichoplax adhaerens* and *Tribolium castaneum* (Figure S1, Table S1). Prior to OrthoMCL comparisons, redundancy was eliminated in each metazoan proteome using the program CD-HIT [34] set to keep only one representative protein (the longest) in clusters of 100% identical proteins. All *Meloidogyne* proteins that clustered with at least another metazoan species in OrthoMCL groups were discarded from the analysis. In root-knot nematode-restricted OrthoMCL groups, only one representative protein sequence per group was kept. The longest *M. incognita* protein was used as reference in all root-knot nematode-restricted OrthoMCL groups except those that contained no *M. incognita* protein but at least one *M. hapla* protein. In these cases, the longest protein from *M. hapla* was selected as a reference.

### BLAST Filtering for Identification of Candidate Lateral Gene Transfers

All predicted proteins that passed the OrthoMCL filter were used as queries for a BLASTp search against a custom database that consisted in the NCBI’s non-redundant (nr) library completed by the whole proteomes of *M. incognita* and *M. hapla*. An e-value cut-off of 0.01 and an alignment covering at least 30% of the query length were required. All proteins that returned at least 50% non-metazoan hits among their ten best blast hits were considered as putatively acquired via lateral gene transfer. The NCBI’s tree of life and Taxonomy IDs associated to protein sequences were used as reference for the taxonomy. To avoid eliminating LGT events that occurred before the separation of different plant-parasitic nematode species or that gave rise to multigene families in these nematodes, BLAST hits that returned Taxonomy IDs corresponding to the three lineage containing plant-parasitic nematodes (Tylenchida: Taxonomy ID: 6300, Triplonchida: Taxonomy ID: 211184 and Dorylaimina: Taxonomy ID: 211225), were not considered in the count of metazoan hits.

### Check for Possible Contaminations

As a gene resulting from bacterial contamination would yield a BLAST result pattern exactly identical to one of a true case of LGT, we further searched and eliminated among the proteins that passed the BLAST filter, those that presented more than 80% sequence identity on more than half of their length (query) with non-metazoan genes.

### Phylogenetic Analyses and Detection of Topologies Compatible with Lateral Gene Transfers

Each *Meloidogyne* protein that passed both the OrthoMCL and the BLAST filters were sent to automated phylogenetic analysis using the FIGENIX [14], [15] platform. Phylogenetic analyses performed with FIGENIX use three different reconstruction methods (neighbor joining, maximum parsimony and maximum likelihood) with bootstrap replications to provide a fusion-tree with support values. Tree topologies corresponding to a potential LGT event were automatically searched among the ensemble of produced phylogenetic trees, using the program PhyloPattern [16]. The pattern searched consisted in the presence of at least a node ‘A’ partitioning the tree in two sub-clades, one monophyletic clade containing only *M. incognita* and/or *M. hapla* and possibly other plant-parasitic nematodes and another distinct clade containing only non-eumetazoan species (any NCBI’s taxid but none descending from 6072, Eumetazoa). Strong phylogenetic support was assigned when an additional node ‘B’ considered as a duplication node and branching to external species was found (Figure 2).

### Functional Annotation of Candidate LGT-acquired Genes

The whole proteomes of *M. incognita* and *M. hapla* were scanned against the Pfam [35] database of HMM protein domains using the PfamScan perl script and the HMMER package. Every root-knot nematode protein sequence was compared to the Pfam-A library (ver. 24.0) of manually curated HMMs using default parameters. Using the Pfam2GO association file, gene ontology terms were assigned to proteins on the basis of their Pfam domain composition. Using the map2slim perl script from the go-perl module, we mapped the initially assigned GO terms to their parent terms in the generic GO-slim ontology. This allowed direct comparison of GO terms at a same granularity level between the different proteins from the two root-knot nematode proteomes, including those originating from LGT.

### Duplications after Transfer Estimated by Phylogenetic Patterns

The number of genes that underwent duplications after transfer was estimated by searching all trees that contained at least two genes from *M. hapla* or at least three genes from *M. incognita* (to correct effects due to the *M. incognita* genome structure in two copies) in the node A subtree corresponding to an LGT event. To detect gene duplications that occurred before the separation of the two lineages, we used PhyloPattern and searched trees that contained both *M. incognita* and *M. hapla* sequences and duplication nodes prior to speciation nodes separating the two species. To detect gene duplications that occurred after the separation of *M. incognita* and *M. hapla* lineages, we searched node ‘A’ subtrees that contained duplication nodes at the base of *M. incognita*-restricted or *M. hapla*-restricted monophyletic groups, using PhyloPattern.

### Lineage-specific Duplications after Transfer Estimated from OrthoMCL Data

We detected in-paralogs or lineage-specific duplications in *M. incognita* and *M. hapla* whole genomes and LGT-acquired genes based on the OrthoMCL analysis conducted with 16 metazoan species. Ever since a gene was present in an OrthoMCL group that contained at least two genes from *M. incognita* or at least two genes from *M. hapla*, the gene was considered as having underwent lineage-specific duplication after the separation of the two nematode species from their last common ancestor. Because a substantial proportion of the genome of *M. incognita* is present in two copies compared to the genome of *M. hapla*, OrthoMCL groups containing two genes from *M. incognita* and a single gene from *M. hapla* were not considered as duplicated.

### Distributions of Transposable Elements and Genes Acquired via LGT

The positions of all gene models on the *M. incognita* scaffolds, including those acquired via LGT, as well as the positions of transposable elements, were retrieved from the GFF files generated at the occasion of the initial annotation of the genome [10]. Annotation of transposable elements (TEs) in the *M. incognita* genome was performed using the REPET pipeline [36]. Using the genome GFF files, we counted the number of TEs in windows of 200, 500, 1,000 and 2,000 bp, flanking LGT genes on the one hand, and flanking the rest of protein-coding genes, on the other hand. Chi^2^ tests were used to compare the distribution of TEs density around LGT genes and the rest of protein-coding genes for the four genomic window sizes.

### Clusters of Genes Acquired via LGT on *M. incognita* Scaffolds

In a same OrthoMCL group, in case several genes from *M. incognita* and/or *M. hapla* were present, we only kept one root-knot nematode gene as representative of the group. Because we were interested in the distribution and enumeration of all potentially laterally acquired genes in the genome of *M. incognita*, we had to take into account the different in-paralogous copies. We assumed that if a representative gene was predicted as potentially resulting from LGT, all the in-paralogous copies resulting from species-specific duplications were equally likely to have been acquired via LGT. We mapped a total of 661 genes, including in-paralogs on the 2,817 *M. incognita* scaffolds. Information about the position of the genes on the different scaffolds was extracted from GFF files generated during the initial annotation of the *M. incognita* genome [10]. We extracted all clusters consisting of at least 3 genes potentially acquired via LGT (including in-paralogs) and distant of less than 50 kb on a same scaffold.

### GC Content and Codon Usage

To measure the GC content and codon usage of protein-coding genes in *M. incognita*, we extracted the corresponding CDS sequences from the GFF files generated at the occasion of the initial annotation of the genome [10]. We used the program geecee from the EMBOSS software package [37] to calculate the GC content of every CDS from predicted LGT gene as well as for the rest of protein-coding genes. We generated codon usage tables for LGT genes and for the rest of protein-coding genes using the cusp program from the EMBOSS package. We then used the codcmp program from EMBOSS to compare codon usage in LGT genes and in the rest of protein-coding genes.

### Mobile Genetic Elements

The 609 non-redundant protein sequences corresponding to genes putatively acquired via lateral gene transfer were compared to the set of proteins present on mobile genetic elements in the ACLAME database [38]. The protein fasta sequences present on known bacterial plasmids, prophages and phages were downloaded from the ACLAME web site (version 0.4, http://aclame.ulb.ac.be). The fasta file containing 122,154 proteins was formatted as a Blast database. The 609 root-knot nematode LGT proteins were searched using BlastP against this library using an e-value cut-off of 0.001. We further filtered nematode proteins that aligned with at least 30% identity on at least 50% of their length and of 50% of the subject length with proteins from the ACLAME database. These proteins were considered as having significant similarities with proteins present on bacterial mobile genetic elements.

## Supporting Information

Figure S1 **Schematic phylogeny of the 16 metazoan species compared at the proteome level.** This tree represent the relative phylogenetic position of the 16 metazoan species compared, including the two root-knot nematodes, *Meloidogyne incognita* and *Meloidogyne hapla*. Besides these two root-knot nematodes, species compared comprise other nematodes (in green), insects (in blue), chordates (in red), urochordates (in orange), cnidaria (in violet) and placozoa (in dark red). Names of the main phylogenetic divisions are given at the corresponding nodes.
(TIF)

Table S1 **List, source and number of proteins in the 16 metazoan species compared.** Names of the 16 metazoan species compared are indicated in the first column, followed by the taxonomic group, the source and version of the proteome retrieved, the number of predicted proteins as well as the number of unique proteins after elimination of redundancy with CD-HIT [34].
(DOC)

Table S2 **Detailed list of the 609 non-redundant root-knot nematode proteins considered as acquired via LGT.** For each protein predicted to have been acquired via LGT of non-metazoan origin, a series of information is listed. Accession numbers, presence of a signal peptide (Y), the number of spliceosomal exons are indicated in columns 1, 2 and 3, respectively. The presence of a Pfam protein domain is indicated in columns 4. Gene Ontology (G.O.) terms associated to the protein based on the Pfam domains composition for the three categories ‘Biological Process’, ‘Molecular Function’ and ‘Cellular Component’, are given in columns 5, 7 and 9, respectively. The G.O. slim terms that were the most over-represented in the set of genes acquired via LGT for the ‘Biological Process’ and ‘Molecular Function’ ontologies are indicated in columns 6 and 8, respectively. The column 10 ‘family’ indicates whether the gene belongs to a known family. If this family corresponds to one previously reported LGT case in the literature (listed in Table 1), the whole line is put in bold. The putative substrate/activity of the protein/enzyme is indicated in column 11. Phylogenetic support (presence of a node ‘A’ or ‘A’ and ‘B’) is given in column 12. If none of the searched phylogenetic patterns were retrieved ‘No’ is indicated in this column whereas in cases no phylogenetic tree at all could be constructed ‘No tree’ is indicated. The last three columns indicate, respectively, the list of species in the donor phylogenetic subtree, the simplified taxonomy of the species found, and the short taxonomy (i.e. Bacteria, Fungi, Protist, Plant or Archae). N/A: not applicable.
(XLS)

Table S3 **Gene Ontology terms assigned to the whole proteomes of root-knot nematodes and to those originating from LGT.** For each Gene Ontology (G.O.) category (A) Biological Process, (B) Molecular Function, (C) Cellular Component, the number of occurrence and abundance (in percent) of G.O. terms are given for the whole *M. incognita* and *M. hapla* proteomes as well as for the proteins considered as acquired via LGT in root-knot nematodes. For comparison purpose, the relative abundance of a G.O. term in proteins acquired via LGT compared to the average abundance observed in the two root-knot nematode is indicated in percent in the last column. Relative abundance follows a heat map color code ranging from red gradient for terms more abundant (+) in LGT proteins to green gradients for terms less abundant (–) in LGT proteins. Yellow gradient indicates a similar abundance of the term in LGT proteins compared to the whole root-knot nematode proteomes.
(XLSX)

Table S4 **Phylogenetically-inferred duplications of LGT genes before and after the separation of the two root-knot nematodes.** This table lists for each of the 141 phylogenetic trees indicating an LGT event, the following information. The accession number of the reference Meloidogyne species used as query for phylogenetic reconstruction. The list of species in the receiver clade. The number of gene copies in *M. hapla* and in *M. incognita*. Whether the gene is observed in one or both the two root-knot nematodes. Whether the gene as underwent duplications either before or after the separation of *M. incognita* and *M. hapla*. Whether the gene as underwent duplications before the separation. Whether the gene as underwent duplications after the separation. The last column indicates the phylogenetic support for the LGT event (‘A’ or ‘A’ + ‘B’).
(XLS)

Table S5 **OrthoMCL-based inference of LGT gene duplications after the separation of the two root-knot nematodes.** This table lists the 123 non-redundant proteins clustered in OrthoMCL groups that contain at least three *M. incognita* proteins or at least two *M. hapla* proteins. Accession numbers of the selected reference proteins are given in the first column. The total numbers of genes from M. incognita and M. hapla in the OrthoMCL group are given in columns 2 and 3, respectively. The total number of root-knot nematode proteins in a given OrthoMCL group and whether a phylogenetic support was assigned is indicated in columns 4 and 5, respectively. Accession numbers of all *M. incognita* and *M. hapla* proteins present in a given OrthoMCL group are listed in the last two columns.
(XLSX)

Table S6 **Genomic clusters of genes acquired via LGT in** ***Meloidogyne incognita.*** This table lists the 161 genes present in 38 genomic clusters composed of at least 3 genes distant of less than 50 kb on a same scaffold. For each gene, the accession number is given, followed that the scaffold and genomic coordinates on the scaffold (columns 1–4). Presence of a Pfam protein domain and grouping in an OrthoMCL group are indicated in columns 5 and 6. In a given genomic cluster, a same OrthoMCL group is represented with a same color. The size (in number of genes) of every genomic cluster is given in the last column.
(XLS)

Table S7 **Putative donors of LGT in root-knot nematodes.** Putative donors for the 141 distinct LGT cases inferred by phylogenetic analysis are listed in this table. Accession numbers of the reference proteins are given in the first column. The nature of the phylogenetic support for LGT is indicated in column 2. Species present in the donor clade are listed in column 3. A simplified taxonomy presenting the clades donor species belong to is given in column 4. Taxonomic division (Bacteria, Fungi, Protist, Plant or Archae) is indicated in the last column.
(XLSX)

Table S8 **LGT proteins with significant hits in bacterial mobile genetic elements.** (A) The list of 146 non-redundant LGT proteins that have a Blast hit <0.01 with mobile genetic elements in ACLAME. First column: accession number, second column: number of exons, third column: best blast hit in ACLAME, last column: whether the protein has a highly significant hit (at least 30% identity on at least 50% of the query length). (B) Details on the 32 LGT proteins that have a highly significant hit in ACLAME. Columns 1–3 are identical to (A). Column 4: description of the ACLAME best blast hit, column 5: the species holding the mobile genetic element, column 6: whether a phylogenetic tree support the LGT event, last column: whether the protein belongs to a family previously described as acquired via LGT in the literature.
(XLSX)

Table S9 **Codon usage of LGT genes and of the rest of protein-coding genes in** ***M. incognita.*** LGT columns show values for genes acquired via LGT whereas “Rest” columns display values for the rest of protein-coding genes. Fraction represents the percent usage of a given codon for a given encoded amino-acid, the sum is thus always = 1 for each amino-acid. Frequency represents the overall average percent usage of a given codon for the whole set of genes (LGT or Rest) the total sum for the 64 codon is = 1. Occurrence: the number of times a codon has been observed in the set of genes acquired via LGT or in the rest of protein-coding genes.
(XLSX)
