# Supplementary material for: Contribution of Lateral Gene Transfers to the Genome Composition and Parasitic Ability of Root-Knot Nematodes
Source: PLoS One. 2012 Nov 30;7(11):e50875. doi: 10.1371/journal.pone.0050875 (PMC3511272; doi:10.1371/journal.pone.0050875)
Supplement: Table S1 — List, source and number of proteins in the 16 metazoan species compared. Names of the 16 metazoan species compared are indicated in the first column, followed by the taxonomic group, the source and version of the proteome retrieved, the number of predicted proteins as well as the number of unique proteins after elimination of redundancy with CD-HIT [34]. (DOC) [file pone.0050875.s002.doc]

**Table S1. The 14 metazoan species compared to *M. incognita* and *M. hapla*.**

| Species name | Taxonomy | Source, version | # of proteins | # of unique proteins^a^ |
| --- | --- | --- | --- | --- |
| *Branchiostoma floridae* | Cephalochordata | V1.0, JGI | 50,817 | 49,325 |
| *Brugia malayi* | Nematoda | V1.0, Wormbase | 11,515 | 11,308 |
| *Bombyx mori* | Insecta | V2008, SilkDB, China | 14,623 | 14,589 |
| *Caenorhabditis briggsae* | Nematoda | WS210, Wormbase | 21,982 | 21,723 |
| *Caenorhabditis elegans* | Nematoda | WS210, Wormbase | 24,273 | 23,855 |
| *Ciona intestinalis* | Urochordata | V2.0, JGI | 14,002 | 13,606 |
| *Drosophila melanogaster* | Insecta | V5.13.56, ENSEMBL | 21,309 | 18,005 |
| *Homo sapiens* | Vertebrata | V37.56, ENSEMBL | 77,748 | 57,177 |
| *Meloidogyne hapla* | Nematoda | V1.0, NC State University | 14,421 | 14,331 |
| *Meloidogyne incognita* | Nematoda | V1.0, INRA / Genoscope | 20,359 | 19,930 |
| *Mus musculus* | Vertebrata | V37.56, ENSEMBL | 40,732 | 35,657 |
| *Nematostella vectensis* | Cnidaria | V1.0, JGI | 27,273 | 26,187 |
| *Pristionchus pacificus* | Nematoda | V1.0, Wormbase | 29,644 | 29,418 |
| *Strongylocentrotus purpuratus* | Echinodermata | V3.0, HGSC, Baylor College of Medicine | 28,944 | 28,533 |
| *Trichoplax adhaerens* | Placozoa | V1.0, JGI | 11,520 | 11,479 |
| *Tribolium castaneum* | Insecta | V2.0, Baylor College of Medicine | 16,422 | 16,357 |

^a^After elimination of redundancy with CD-HIT clustering at 100% identity.
